# Large-scale ligand-based predictive modelling using support vector machines

**DOI:** 10.1186/s13321-016-0151-5

**Published:** 2016-08-10

**Authors:** Jonathan Alvarsson, Samuel Lampa, Wesley Schaal, Claes Andersson, Jarl E. S. Wikberg, Ola Spjuth

**Affiliations:** 1 Department of Pharmaceutical Biosciences, Uppsala University, 751 24 Uppsala, Sweden; 2 Science for Life Laboratory, Uppsala University, 751 24 Uppsala, Sweden; 3 Department of Medical Sciences, Uppsala University, 751 85 Uppsala, Sweden

**Keywords:** Predictive modelling, Support vector machine, Bioclipse, Molecular signatures, QSAR

## Abstract

The increasing size of datasets in drug discovery makes it challenging to build robust and accurate predictive models within a reasonable amount of time. In order to investigate the effect of dataset sizes on predictive performance and modelling time, ligand-based regression models were trained on open datasets of varying sizes of up to 1.2 million chemical structures. For modelling, two implementations of support vector machines (SVM) were used. Chemical structures were described by the signatures molecular descriptor. Results showed that for the larger datasets, the LIBLINEAR SVM implementation performed on par with the well-established libsvm with a radial basis function kernel, but with dramatically less time for model building even on modest computer resources. Using a non-linear kernel proved to be infeasible for large data sizes, even with substantial computational resources on a computer cluster. To deploy the resulting models, we extended the Bioclipse decision support framework to support models from LIBLINEAR and made our models of logD and solubility available from within Bioclipse.

**Electronic supplementary material:**

The online version of this article (doi:10.1186/s13321-016-0151-5) contains supplementary material, which is available to authorized users.

## Background

Ligand-based predictive modelling is widely used in drug discovery, primarily in lead identification, optimization, and safety assessment [1–3]. A common ligand-based method is quantitative structure-activity relationship (QSAR), where molecular properties are modelled by numerically describing the molecules and correlating the numerical description to the molecular properties [4]. Such QSAR models can then be used for predicting properties for new, unknown compounds with common examples including toxicity, biological activity, and physicochemical properties.

Datasets useful for ligand-based predictive modelling are increasing in size and number, partly due to high-throughput in vitro technologies and the accumulation of data in public repositories. Increasingly larger datasets provide new challenges to build robust and accurate predictive models within a reasonable amount of time, and may require the use of high-performance computing (HPC) or cloud computing resources [5].

Apart from the time and cost of building models on large datasets there are also challenges for delivering the resulting models to the users involved in drug discovery projects. A common way to deliver ligand-based models is to deploy them as Web services, which can be consumed by users by submitting a chemical structure (within a Web page or a third party application) that is transferred over a network to the Web service where the prediction is carried out, and the result is then returned via the network. Another approach is to make predictions on the user’s local computer. This has the advantage of avoiding transferring potentially sensitive chemical structures over a network.

In this project we set out to study the task of building QSAR models on very large datasets. Publicly available datasets are commonly limited in size. We identified a dataset of measured solubility from which we extracted about 37 thousand substances [6]. In order to study larger datasets we used data from the ChEMBL [7] database which contains calculated properties. We selected the molecular property logD and extracted more than a million substances with this property calculated.

Support vector machines (SVM) is commonly used for building QSAR models as reported by, for example, Darnag et al. [8]. SVM has also, together with substructure fingerprints, been successfully used for predicting the LogD-related value LogP [9]. In this work we described chemical structures with the signature descriptor [10] which has been shown to produce good results together with SVM for QSAR predictions [11–13] and lately was used for identifying Cathepsin-L inhibitors [14]. We used open data from ChEMBL and a public dataset of measured solubility values to train QSAR models using SVM. The resulting models were studied with regard to the effect of varying the training set size both with regards to training time as well as prediction performance, and the models were made available from the Bioclipse platform [15, 16] via its decision support functionality [17].

## Methods

### Data

Solubility, the concentration of a dissolved compound in equilibrium with a solvent, is a fundamental physicochemical property. We used a dataset [6] originally containing 57,859 compounds but with some values only tabulated as larger than a cut-off value. In order to avoid complications with modelling ‘larger than’ relations, we removed all entries with inequalities and thereby ended up with 37,099 solubility data points. We modelled the logarithm of the values, which made the solubility dataset similar to the LogD dataset.

ChEMBL is an open data chemical database containing more than one million compounds, manually curated with data extracted from the chemical literature and with calculated molecular properties appended [7, 18, 19]. From ChEMBL version 17 we extracted all substances having the calculated property acd_logd (logD) at pH = 7.4, resulting in 1,270,472 substances.

LogP is an estimate of lipophilicity, which is an important property in drug discovery as it relates to cell membrane penetration [20]. Specifically, logP is the log of the value *P*, which is the partitioning of the neutral form of a compound between immiscible phases of octanol and water. LogD is logP with the consideration of ionized forms of the compound at a defined pH. A pH of 7.4, which is the average value for human blood, is commonly used for logD.

### Signature molecular descriptor

**Fig. 1:**
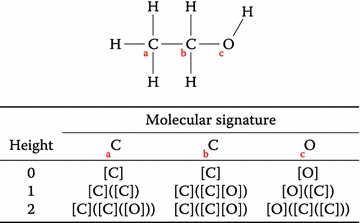
Signatures of height 0–2 for ethanol. *Note* that hydrogen atoms are not included in the signatures. The ethanol molecule is so small that increasing the signature height beyond height 2 makes no difference

The signature molecular descriptor [10] is a descriptor made up of atom signatures calculated for the atoms of a molecule, where an atom signature consists of a canonical description of the environment around the atom with its size controlled by a height parameter (see Fig. 1 for an example showing the signatures of ethanol). Larger heights mean higher information content, but more information also requires more memory and computational power when building predictive models.

We described chemical structures with molecular signatures and used a combination of consecutive heights 1–3, i.e., an atom distance of up to 3 atoms; values which have previously been shown to produce good results for SVM modelling [21]. We used the molecular signatures implementation in the open source cheminformatics library Chemistry Development Kit (CDK) [22, 23] version 1.5.7.

### QSAR modelling

For modelling we used support vector machines [24], a machine learning method that has been used extensively in predictive modelling in cheminformatics [25, 26]. The algorithm can use a kernel function to map the problem into a high dimensional space where the problem can be easier to solve. The radial basis function (RBF) kernel performs this mapping in a non-linear fashion. It is a commonly used kernel that has been suggested as a good starting point for SVM modelling [27] and has previously been successfully used in QSAR studies [5, 17, 21]. *π*SVM [28] is a software implementation which enables distributed SVM calculations over multiple computation nodes of a computer cluster, which facilitates training SVM models on large datasets. SVM with the RBF kernel has two parameters which need to be determined, *cost* and *γ*. The *cost* parameter limits over-fitting and the *γ* parameter affects the RBF-kernel. When tuning SVM parameters in this study we started with a grid search on a sample of our dataset to find good values of *cost* and *γ* for regression.

We also tested linear SVM using the implementation in the LIBLINEAR software [29], which does not support parallel execution. Linear SVM comes with one parameter, *cost*, and we used a cross-validated parameter search on the training set to determine a good *cost* value for LIBLINEAR.

**Fig. 2:**
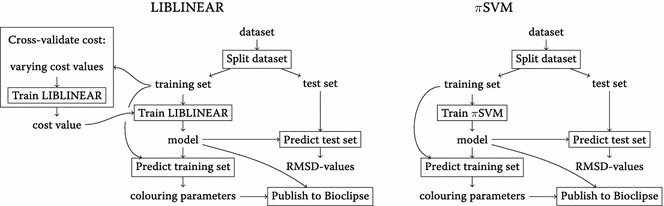
Workflow diagrams. Diagrams showing the workflows for the LIBLINEAR and *π*SVM modelling

Figure 2 shows workflow diagrams for the LIBLINEAR and *π*SVM modeling.

*π*SVM models were built using four computer nodes, each consisting of two 8-core CPUs (Intel Xeon E5-2660, HP ProLiant SL230s Gen8), for a total of 64 cores. Each node had a memory configuration of 128 GB. The LIBLINEAR models were built using one such core (i.e., 8 GB of memory).

### Study design

We studied two datasets, one with measured solubility data and one with calculated logD data. For each dataset, two factors were varied in the study: training set size (*N*) and machine learning method (*M*). The values for the factors used are tabulated in Table 1.

In order to decide on *cost* and *γ* for SVM RBF we used data from the logD dataset; a training set of 5000 chemical structures together with a test set of 50,000 structures, and evaluated the predictive performance of the models for varying *cost* and *γ*. The best performing combination of *cost* and *γ* was chosen and the structures used for determining these factors were removed and not used in the subsequent analysis. Performing a cross-validated grid search on the training set for SVM RBF was judged as infeasible because of the excessive execution time. In the case of LIBLINEAR, the execution times were so much smaller that we could use a cross-validated parameter search on the training set to find *cost* values. Many *cost*-values for the linear SVM resulted in the same performance for the same training set size. In these cases we favoured lower *cost* values, which is an optimistic choice.

**Table 1:** Training set sizes tested during the study for the different methods and datasets

Linear SVM		RBF SVM	
Solubility	logD	Solubility	logD
100	100	100	100
1000	1000	1000	1000
5000	5000	5000	5000
20,000	20,000	20,000	20,000
32,096	40,000	32,096	40,000
	80,000		80,000
	160,000		160,000
	320,000		
	1,188,343

**Table 2:** Costs chosen by the cross-validation for linear SVM using LIBLINEAR for the different training set sizes

Solubility		logD	
Training set size	Found cost	Training set size	Found cost
100	100,000	100	10,000
	2		0.01
	0.1		0.005
1000	0.05	1000	10,000,000
	0.05		1000
	0.05		0.1
5000	0.05	5000	0.5
	0.05		0.75
	0.05		0.1
10,000	0.05	10,000	100
	0.05		2
	0.05		0.25
20,000	0.1	20,000	0.5
	0.1		0.5
	0.1		1
32,096	0.1	80,000	0.5
	0.1		0.5
	0.1		0.75
		160,000	0.75
			0.5
			0.5
		320,000	0.75
			0.5
			0.5
		1,188,343	0.5
			0.5
			1

Note the highly variable results among the three replicates for the small dataset sizes and low variation among the replicates for the larger training set sizes

### Model provisioning via Bioclipse

Bioclipse is a workbench for the life sciences that provides open source drug discovery functionality [30]. Bioclipse decision support (DS) [17] provides a framework for making predictive models available to end users running on a local computer (off-line). The users can, through the graphical user interface, download and install predictive models which can be executed for single molecules as well as on collections of molecules. The predicted results can be visually interpreted, as the signature that contributed the most to the prediction can be shown as a set of coloured atoms in the user interface [26, 31].

Running predictive models on a local computer has the advantage that users are not dependent on a network connection for predictions with no risk for delays due to unresponsive remote servers. Another advantage is that no chemical information is sent over the network (as is the case when predictive models are provisioned as Web services). However, for an off-line predictive system with multiple large models, the size of models can become an issue, as they need to be downloaded and used on a local computer.

When predicting molecular properties using Bioclise DS, the molecular signatures for the query structure are calculated. In the SVM model these signatures are represented as a vector of integers corresponding to a list of the signatures that were found in the query structure. In order for Bioclipse to be able to produce this vector of integers, the SVM model file comes with another file listing all signatures used when training the model. These two files need to be read into memory by Bioclipse and for large training sets these files may be large. Users may work with 50 or even 100 models at the same time, which means that the trade-off between the model’s size and performance can become important even on today’s computers.

### Graphing and statistics

Plots and statistics were made using the statistical programming language R [32].

## Results

**Fig. 3:**
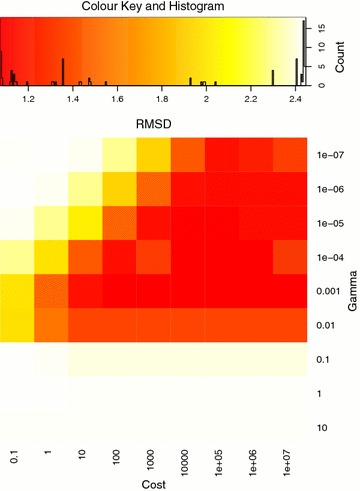
Heatmap of RMSD from the grid search. Above the heatmap is a colour key and histogram. Different *cost* and *γ* values were tested using a training set size of 5000 and a test set size of 50,000. The heat map was made using the R package gplots [34]

A grid search on a small subset of the logD dataset was performed to determine the SVM-RBF parameters *cost* and *γ*. A heat map generated from the grid search for these parameters is available in Fig. 3. The best performing combination in the grid search was *cost* = 100 and *γ* = 0.001 and these parameters were used in the *π*SVM runs. For the linear SVM, a cross-validated parameter search was performed to determine *cost*. Table 2 lists the resulting optimal *cost* values for the two datasets and the various training set sizes. We note that as the training set size increases, the stability among the values from the cross-validation also increases. For training set size 100, the *cost* values range from 0.005 to 10,000 but for the larger training set sizes they seem to stabilize around 0.1 for the solubility dataset and around 0.5 to 1 for the logD dataset.

**Fig. 4:**
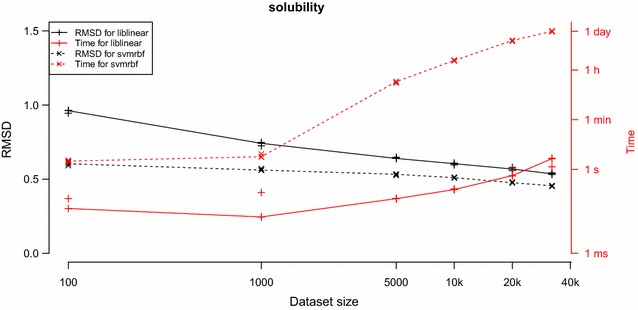
Learning curves and model creation time for the solubility dataset. The *plot* shows learning curves (in *black*) overlaid with curves for model creation times (in *red*) for varying dataset sizes and the two SVM implementations. Plotted are measured values with lines drawn between the medians. *π*SVM was run on 64 cores on the cluster and LIBLINEAR was run using one such core. LIBLINEAR was much faster but even though the two *black curves* seem to converge there is a difference between them that in many cases probably is relevant, especially so at these small dataset sizes

**Fig. 5:**
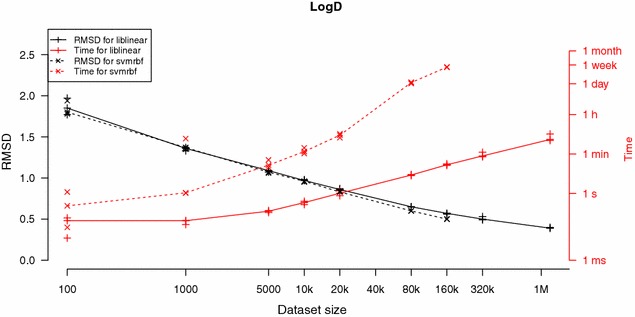
Learning curves and model creation time for the logD dataset. The *plot* shows learning curves (in *black*) overlaid with curves for model creation times (in *red*) for varying dataset sizes and the two SVM implementations. Plotted are measured values with lines drawn between the medians. *π*SVM was run on 64 cores on the cluster and LIBLINEAR was run using one such core. LIBLINEAR was much faster and gave similar performance in RMSD for the same training set sizes as *π*SVM

**Fig. 6:**
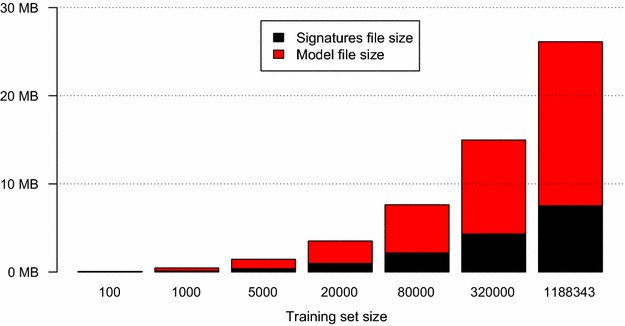
File sizes. The sizes (in MB) of the signatures list file and the model file from LIBLINEAR for different training set sizes of the logD dataset

**Fig. 7:**
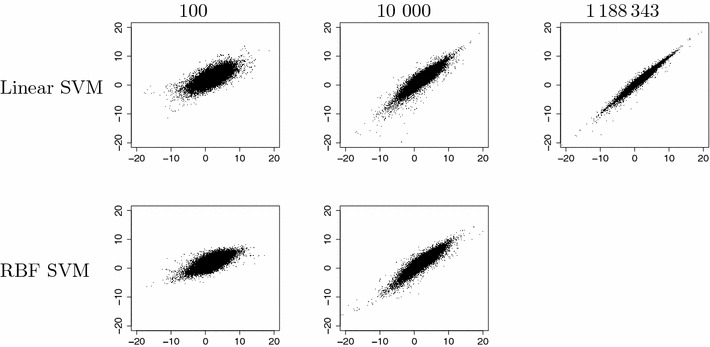
Predicted versus original values of logD. Predicted values (*y-axis*) versus original values (*x-axis*) for a few representative training set sizes of the logD dataset and the the two variants of SVM used (Linear and RBF)

**Fig. 8:**
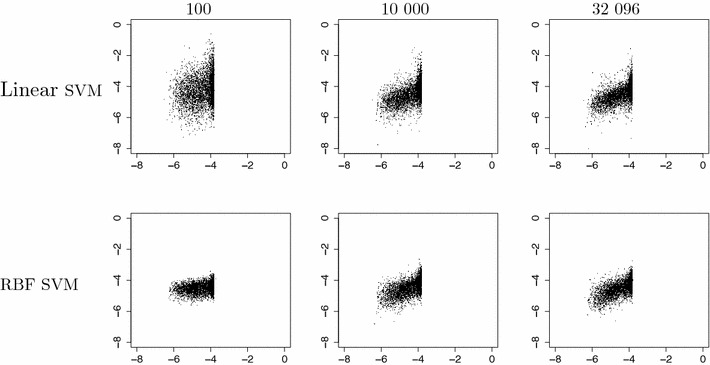
Predicted versus original values of solubility. Predicted values (*y-axis*) versus original values (*x-axis*) for a few representative training set sizes of the solubility dataset and the the two variants of SVM used (Linear and RBF)

We trained models with linear SVM and SVM-RBF on varying dataset sizes from our two tested datasets according to the study design. Figures 4 and 5 show learning curves and model build time for the tested SVM implementations and training set sizes for the two datasets, respectively. The sizes of the SVM model file and the signatures file for different training set sizes for the LIBLINEAR approach are plotted in Fig. 6. Figures 7 and 8 show predicted versus original values for the logD and solubility datasets, respectively. A similar plot for the solubility dataset where the prediction was made using ChemAxon’s solubility predictor [33] is included as Additional file 1.

**Fig. 9:**
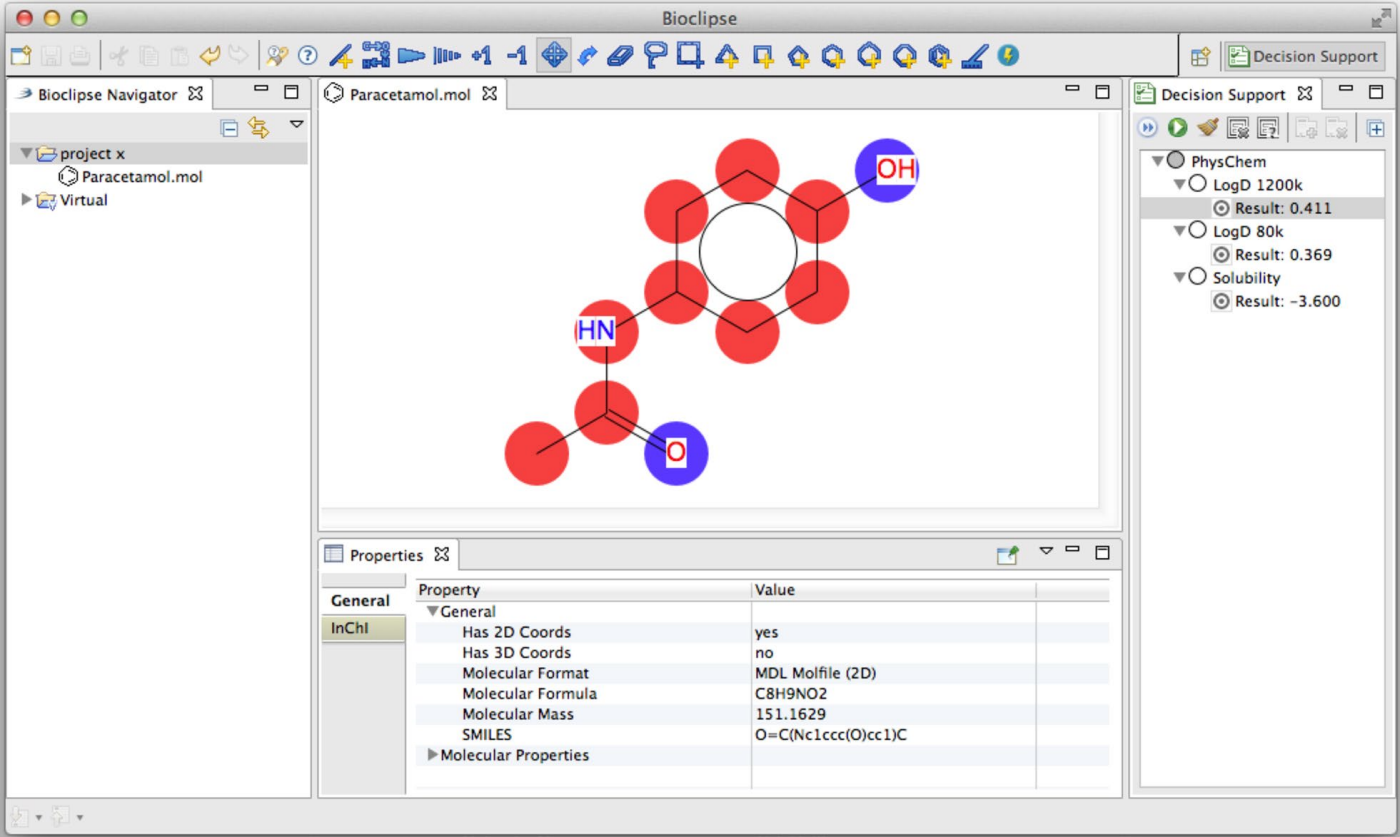
Bioclipse screenshot. The produced models were published to Bioclipse DS. Atoms marked *red* in the figure have contributed more to the prediction, while atoms marked *blue* have contributed less

Bioclipse DS was extended to handle LIBLINEAR models and the trained models are planned to be included in the next Bioclipse release. Figure 9 contains a screenshot of Bioclipse DS running the produced models. For instructions on how to install them in the meantime see: http://wiki.bioclipse.net/index.php?title=MM-Models.

## Discussion

We observe that the linear SVM of LIBLINEAR was dramatically faster than the RBF SVM implementation in *π*SVM. Even though *π*SVM was run on 64 cores, it was not feasible to run it on the larger training sets constructed from the logD dataset; the largest dataset we were able to model consisted of 160,000 substances. When running on the shared computer cluster, it is generally desired that jobs should finish within a week. Running *π*SVM on 160,000 substances using 64 cores took more than 5½ days which corresponds to more than 8500 core hours.

In an earlier study we benchmarked the effect of varying *cost* and *γ* when doing classification using the SVM and the RBF kernel with the result that a sweet spot is located around *cost* = 10 and *γ* = 0.01 [21]. We find it promising that the best combination of *cost* and *γ* for RBF SVM in this study was so close in the grid that, in fact, with a slightly different division of the tested values it seems likely that we would have gotten the exact same *cost* and *γ* combination also for the regression problem in this study.

When determining the *cost* and *γ* for *π*SVM we used a sample of our logD dataset to do a grid search, and then we removed that data from the subsequent analysis. An alternative would have been to do a cross-validation. However, building the RBF SVM models would have taken too long and removing a small part of the data in this case does not have a major impact on the results as data was abundant.

The LIBLINEAR method was used for the final model for the logD dataset and the SVM RBF method was used for the solubility dataset. The 80 k logD dataset resulted in files of less than 8 MB as can be seen in Fig. 6 but it had a somewhat high RMSD of around 0.65 log units. The 1.2 million dataset gave an RMSD of around 0.4 log units but had a file size of around 27 MB. We decided to publish both the small and the large models for logD and let the user choose whether they want the higher accuracy or the model with a smaller file size.

For logD it is our opinion that the difference in prediction performance for the two methods is so small that the vast difference in execution time motivates the use of LIBLINEAR over *π*SVM at least for the larger training set sizes. Also for the solubility dataset there is a large difference in execution time between the models but, although seemingly decreasing as training size increases, we also note a gap in prediction performance that might be of relevance. This difference in performance is also visible when comparing linear SVM with RBF SVM in Fig. 8. It can be worthwhile to use an RBF kernel and get a slightly more accurate model for the smaller dataset sizes, i.e., when a user can afford the time to wait for a model to build over night instead of in a few seconds.

## Conclusion

Our study shows that when using large datasets the choice of machine learning method becomes important. The linear SVM in LIBLINEAR produces models with similar predictive performance (for larger training set sizes) as the RBF SVM approach in *π*SVM, but with dramatically lower modelling time. For smaller dataset sizes we see some indications that *π*SVM might perform better but as datasets get bigger the less approximative method of RBF SVM becomes too slow to be a reasonable alternative. Even when using parallel computation on 64 cores, *π*SVM demanded so much computational power that we could not build the models for the largest datasets.

Using LIBLINEAR it was possible to build regression QSAR models based on over one million substances. Bioclipse DS allowed us to make such models available through a point and click interface, and with visual interpretation consisting of highlighted chemical substructures (highlighting what contributed the most to the predicted result). With our choice to include both small and large model versions, users can choose between a model with maximal predictive performance or a smaller model with slightly less predictive performance.

## Additional file

10.1186/s13321-016-0151-5 Details and plots for the solubility dataset.

## References

[1] Cumming JG, Davis AM, Muresan S, Haeberlein M, Chen H (2013). Chemical predictive modelling to improve compound quality. Nat Rev Drug Discov.

[2] Muster W, Breidenbach A, Fischer H, Kirchner S, Miiller L, Pahler A (2008). Computational toxicology in drug development. Drug Discov Today.

[3] Raunio H (2011). In silico toxicology – non-testing methods. Front Pharmacol.

[4] Hansch C (1969). Quantitative approach to biochemical structure-activity relationships. Acc Chem Res.

[5] Moghadam BT, Alvarsson J, Holm M, Eklund M, Carlsson L, Spjuth O (2015). Scaling predictive modeling in drug development with cloud computing. J Chem Inf Model.

[6] National Center for Biotechnology Information. PubChem BioAssay Database; AID = 1996. https://pubchem.ncbi.nlm.nih.gov/bioassay/1996

[7] Gaulton A, Bellis LJ, Bento AP, Chambers J, Davies M, Hersey A, Light Y, McGlinchey S, Michalovich D, Al-Lazikani B (2012). Chembl: a large-scale bioactivity database for drug discovery. Nucleic Acids Res.

[8] Darnag R, Mazouz EM, Schmitzer A, Villemin D, Jarid A, Cherqaoui D (2010). Support vector machines: development of QSAR models for predicting anti-HIV-1 activity of TIBO derivatives. Eur J Med Chem.

[9] Liao Q, Yao J, Yuan S (2006). SVM approach for predicting logP. Mol Divers.

[10] Faulon J-L, Visco DP, Pophale RS (2003). The signature molecular descriptor. 1. Using extended valence sequences in QSAR and QSPR studies. J Chem Inf Comput Sci.

[11] Norinder U, Ek ME (2013). QSAR investigation of NaV1. 7 active compounds using the SVM/signature approach and the bioclipse modeling platform. Bioorg Med Chem Lett.

[12] Spjuth O, Georgiev V, Carlsson L, Alvarsson J, Berg A, Willighagen E, Wikberg JE, Eklund M (2013). Bioclipse-R: integrating management and visualization of life science data with statistical analysis. Bioinformatics.

[13] Alvarsson J, Eklund M, Engkvist O, Spjuth O, Carlsson L, Wikberg JES, Noeske T (2014). Ligand-based target prediction with signature fingerprints. J Chem Inf Model.

[14] Chen JJF, Visco DP (2016). Developing an in silico pipeline for faster drug candidate discovery: Virtual high throughput screening with the signature molecular descriptor using support vector machine models. Chem Eng Sci.

[15] Spjuth O, Helmus T, Willighagen EL, Kuhn S, Eklund M, Wagener J, Murray-Rust P, Steinbeck C, Wikberg JE (2007). Bioclipse: an open source workbench for chemo-and bioinformatics. BMC Bioinform.

[16] Spjuth O, Alvarsson J, Berg A, Eklund M, Kuhn S, Masak C, Torrance G, Wagener J, Willighagen EL, Steinbeck C (2009). Bioclipse 2: a scriptable integration platform for the life sciences. BMC Bioinform.

[17] Spjuth O, Eklund M, Ahlberg Helgee E, Boyer S, Carlsson L (2011). Integrated decision support for assessing chemical liabilities. J Chem Inf Model.

[18] Overington J (2009) ChEMBL. An interview with John Overington, team leader, chemogenomics at the European Bioinformatics Institute Outstation of the European Molecular Biology Laboratory (EMBL-EBI). Interview by Wendy A. Warr. Springer, Heidelberg 10.1007/s10822-009-9260-9 19194660

[19] Papadatos G, Overington JP (2014). The chEMBL database: a taster for medicinal chemists. Future Med Chem.

[20] Waring MJ (2010). Lipophilicity in drug discovery. Exp Opin Drug Discov.

[21] Alvarsson J, Eklund M, Andersson C, Carlsson L, Spjuth O, Wikberg JES (2014). Benchmarking study of parameter variation when using signature fingerprints together with support vector machines. J Chem Inf Model.

[22] Steinbeck C, Han Y, Kuhn S, Horlacher O, Luttmann E, Willighagen E (2003). The chemistry development kit (CDK): an open-source Java library for chemo-and bioinformatics. J Chem Inf Comput Sci.

[23] Steinbeck C, Hoppe C, Kuhn S, Floris M, Guha R, Willighagen EL (2006). Recent developments of the chemistry development kit (CDK)—an open-source Java library for chemo-and bioinformatics. Curr Pharm Des.

[24] Smola AJ, Scholkopf B (2004). A tutorial on support vector regression. Stat Comput.

[25] Burbidge R, Trotter M, Buxton B, Holden S (2001). Drug design by machine learning: support vector machines for pharmaceutical data. Comput Chem.

[26] Carlsson L, Helgee EA, Boyer S (2009). Interpretation of nonlinear QSAR models applied to Ames mutagenicity data. J Chem Inf Model.

[27] Hsu C-W, Chang C-C, Lin C-J (2009) A practical guide to support vector classification. http://www.csie.ntu.edu.tw/cjlin/papers/guide/guide.pdf

[28] PiSvM Software. http://pisvm.sourceforge.net. Accessed 26 Mar 2015

[29] Fan R-E, Chang K-W, Hsieh C-J, Wang X-R, Lin C-J (2008). Liblinear: a library for large linear classification. J Mach Learn Res.

[30] Spjuth O, Carlsson L, Alvarsson J, Georgiev V, Willighagen E, Eklund M (2012). Open source drug discovery with bioclipse. Curr Topics Med Chem.

[31] Ahlberg E, Spjuth O, Hasselgren C, Carlsson L, Gammerman A, Vovk V, Papadopoulos H (2015). Interpretation of conformal prediction classification models. Statistical learning and data sciences: Third international symposium, SLDS 2015, Egham, UK, April 20–23, 2015, proceedings.

[32] R Core Team (2014) R: a language and environment for statistical computing. R Foundation for Statistical Computing, Vienna, Austria. http://www.R-project.org/

[33] Calculator Plugins version 15.11.2.0, ChemAxon. http://www.chemaxon.com

[34] Warnes GR, Bolker B, Bonebakker L, Gentleman R, Liaw WHA, Lumley T, Maechler M, Magnusson A, Moeller S, Schwartz M, Venables B (2014) Gplots: various R programming tools for plotting data. R package version 2.14.0. http://CRAN.R-project.org/package=gplots

