# Supplementary material for: Large-scale ligand-based predictive modelling using support vector machines
Source: J Cheminform. 2016 Aug 10;8:39. doi: 10.1186/s13321-016-0151-5 (PMC4980776; doi:10.1186/s13321-016-0151-5)
Supplement: Supplementary file 1 — 10.1186/s13321-016-0151-5 Details and plots for the solubility dataset. [file 13321_2016_151_MOESM1_ESM.pdf]

Additional material to 'Large-scale ligand-based predictive modelling using support vector machines'

This supplemental material comprise details and plots for the solubility dataset. The dataset is quite skewed as can be seen in Figure 1. In order to get a comparison of another way of predicting solubility we used a solubility predictor from ChemAxon[1]. Figure 2 shows the results of solubility prediction of 5 000 random molecules from the dataset using ChemAxons solubility predictor.

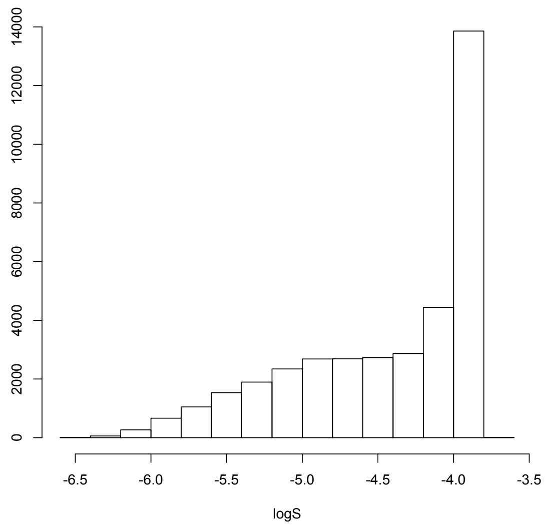

Figure 1: Histogram showing the distribution of the solubility values in the solubility dataset.

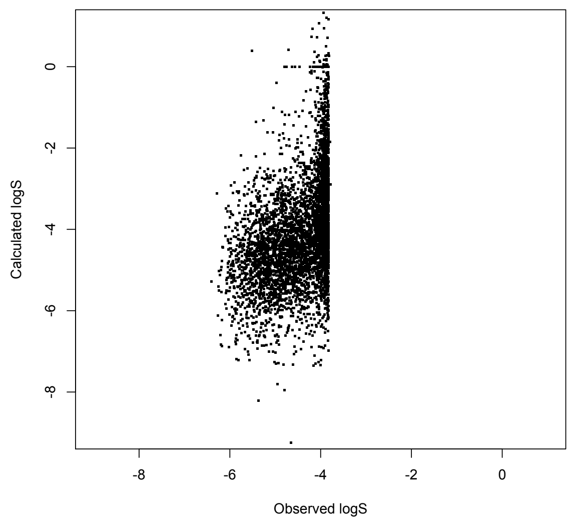

Figure 2: Solubility calculated using ChemAxon plotted against measured solubility for 5 000 molecules randomly chosen from the solubility dataset

[1] Calculator Plugin, version 15.11.2.0, ChemAxon, `http://www.chemaxon.com`
